# Expression of Cyr61 is associated with clinical course in patients with Crohn’s disease

**DOI:** 10.1186/s12876-021-01713-9

**Published:** 2021-03-20

**Authors:** Su-Mi Lee, Kyung-Hwa Lee, Seon-Young Park, Dong Hyun Kim, Jin Ook Chung, Jae Kyun Ju, Jae-Hyuk Lee, Hyun Soo Kim

**Affiliations:** 1 Department of Internal Medicine, Chonnam National University Medical School, 42, Jaebongro, Dong-ku, Gwangju, 501-757 Korea; 2 Department of Pathology, Chonnam National University Medical School, Gwangju, South Korea; 3 Department of General Surgery, Chonnam National University Medical School, Gwangju, South Korea

**Keywords:** Crohn’s disease, Cyr61, Inflammation, Recurrence

## Abstract

**Backgrounds:**

Cysteine-rich angiogenic inducer 61 (Cyr61) is emerging as an important regulator of tissue homeostasis and wound repair. We aim to explore the colonic mucosal expression of Cyr61 and analyze the association between Cyr61 expression and clinical course in patients with Crohn’s disease (CD).

**Methods:**

Endoscopic samples were identified from 83 CD patients with and 372 controls by searching pathological reports. Among them, age- and sex- matched 43 of each group by a propensity score were selected to compare Cyr61 expression by immunohistochemistry (IHC). IHC scores for Cyr61 expression of CD patients were divided into tertiles to evaluate the association with clinical course. We also measured the level of mRNA for Cyr 61 and proinflammatory genes in inflamed and noninflamed colonic mucosal lesions from CD patients.

**Results:**

The mean IHC scores for Cyr61 expression was higher in CD patients (86.5) than in controls (46.1, *P* < 0.001). In CD patients, the mean IHC scores for Cyr61 expression (68.3) was lower in patients with clinical recurrence than in patients without recurrence (92.2, *P* = 0.01). Cyr61 mRNA levels in inflamed mucosa were twofold higher than those in non-inflamed lesion (*P* > 0.05) and the mRNA levels of IL-6 and TLR-4 in inflamed mucosa were significantly higher than those in non-inflamed mucosa in CD patients (all *P* < 0.05). When CD patients were stratified into tertile groups according to IHC scores for Cyr61 expression, clinical recurrence rates tended to be lower in patients with high Cyr61 expression (*P* for trend = 0.02). Compared with tertile 1 of Cyr61 expression, tertile 3 of Cyr 61 expression was associated with reduced risk of clinical recurrence (OR 0.43, 95% CI 0.20–0.92) after adjustment for age, sex and CD activity index at the time of colonoscopy in CD patients (*P* = 0.03).

**Conclusions:**

Cyr61 mucosal expression in CD patients was inversely associated with clinical course. Future study need to be considered to evaluate whether Cyr 61 may play a role in activating inflammatory responses and contributing to wound healing and tissue repair in patients with CD.

## Background

Crohn’s disease (CD) is characterized by idiopathic chronic inflammatory damage affecting any portion of the intestinal tract [1]. To preserve normal homeostasis, wound-healing processes following injuries or physiological damage are needed [2], and better understanding of these repair mechanisms may aid treatment approaches for damaged intestine in patients with CD.

Cysteine-rich angiogenic inducer 61 (Cyr61, CCN1) is a secreted heparin-binding extracellular matrix-associated protein and is emerging as an important regulator of tissue homeostasis and wound repair through the control of cell adhesion and cell migration [3, 4]. Previous study showed that Cyr 61 levels in the colonic mucosa from patients with inflammatory bowel disease and mice with experimental colitis were increased, suggesting the involvement of Cyr61 in the pathogenesis of a colitis model [5]. Other study demonstrated lower Cyr61 expression in dermal fibroblasts from patients with systemic sclerosis compared to healthy controls [6]. Recently, Lin et al. suggested that serum Cyr61 was associated with inflammatory cytokines and disease activities in patients with rheumatoid arthritis and systemic lupus erythematosus (SLE) [7, 8]. Until now, there is limited information regarding whether Cyr61 plays any role in inflammatory processes or is associated with clinical disease activity and/or clinical course in patients with CD.

In this study, we aim to explore the colonic mucosal expression of Cyr61 in patients with CD and analyze the association between Cyr61 expression and clinical disease activity and/or prognosis.

## Methods

### Subjects and samples

We identified endoscopic biopsy samples from ileocolic lesion of 83 patients with CD and 372 controls by searching pathological report files at Chonnam National University Hospital between Jan 2018 and June 2019. All 372 control group subjects had no significant pathological findings and no clinical history of inflammatory bowel disease or neoplasia. Among 83 CD patients and 372 controls, 43 of each group were selected through propensity score matching to reduce the effects of age and sex.

### Histopathologic assessment of inflammatory activities and immunohistochemistry of Cyr61

To evaluate the histopathologic assessment of inflammatory activities of biopsy specimens in patients with CD, we used the pathologic scoring system suggested by Naini et al. [9] including ileitis score (0–10) and colitis score (0–17).

Cyr61 protein expression in mucosal tissues was evaluated by immunohistochemistry (IHC). Briefly, formalin-fixed paraffin-embedded (FFPE) blocks were cut at 3-μm thickness and immunostained with a specific antibody against Cyr61 protein (1:1500 dilution; catalog no. ab10760; Abcam, Cambridge, UK) using an automated immunostainer (Bond-maX DC2002; Leica Biosystems, Bannockburn, IL, USA). For antigen retrieval, programmed heat-induced epitope retrieval was carried out using bond epitope retrieval solution 1 (containing citrate buffer at pH 6.0) for 15 min. IHC slides were assessed by two experienced pathologists (JHL and KHL), who were blinded to the clinical details. Immunohistochemical staining was re-evaluated for cases showing disagreement between observers. Two pathologists reviewed the cases together and then reached an agreement for inconclusive samples.

The intensity of cytoplasmic immunoreactivity was initially classified into four grades: no staining, weak positivity, moderate positivity, and strong positivity. No cases were completely immunonegative for Cyr61. Cases with weak staining intensity were categorized as ‘low-expression’ and those with moderate or strong staining intensity were considered as ‘high-expression’. The proportion of the stained area was estimated by the ratio of positively stained area over the whole area and expressed as a percentage. IHC scores for Cyr61 expression were calculated by multiplying the intensity grade scale by the stained area percentage [10].

Microscopy images were acquired on a Nikon Eclipse 80i microscope with Plan Fluor objective lenses (DIC M/N1 × 10 and DIC M/N2 × 40) using a Nikon DS-Ri2 camera and imaging software NIS-Elements F Ver4.30.01 (Nikon corporation, Tokyo, Japan). The original images were taken with both Fast (Focus) and Quality (Capture) settings set to 3 × 8bit 1636 × 1088 and then transformed from combination of width 57.71 cm, height 38.38 cm and resolution 72 dpi to that of width 6.0 cm, height 4.0 cm and resolution 600 dpi using Adobe Photoshop version 22.1.1 (Adobe, San Jose, CA, USA).

### RNA isolation and real-time polymerase chain reaction of pro-inflammatory genes and Cyr61

In our previous study, we compared the mRNA levels of proinflammatory genes in the colonic paired mucosal biopsy specimens (inflamed and noninflamed lesions) from each 32 patient with CD [11]. In this study, we used the RNA remaining from those samples to evaluate the degree of inflammation and Cyr61 expression in paired samples from each patient. As we did not have enough amount of RNA in 21 patients, we could analyze the mRNA levels from only 11 patients with CD. Total RNA was extracted using Trizol (Takara, Tokyo, Japan). Briefly, 1 mL of Trizol solution was added into each well, and then the suspension was collected into a 1.5 mL tube. After adding 200 μL of chloroform (Sigma-Aldrich, St. Louis, MO, USA) and vortexing for 15 secs, the mixture was centrifuged at 20,000×*g* for 20 min. The supernatant was then collected and mixed with equal amounts of isopropyl alcohol (MERCK, Kenilworth, NJ, USA) followed by centrifugation at 20,000×*g* rpm for 20 min. The pellet was washed with 1 mL of 70% ethyl alcohol (MERCK) and centrifuged at 20,000×*g* for 5 min. After removing the remaining ethyl alcohol, the RNA pellet was air dried at room temperature and then suspended in 50 μL of diethyl pyrocarbonate water.

### Outcomes

We retrieved the clinical disease activities score such as Crohn’s disease activity index (CDAI), which was calculated and written on the chart by colonoscopists (KHS and KDH) from the collection of daily status for past 7 days around the time of colonoscopy. We also retrieved endoscopic disease activities scores such as simple endoscopic score of Crohn’s disease (SES-CD) scale and Crohn’s disease endoscopic index of severity (CDEIS) from the medical records, which were calculated by experienced colonoscopists (KHS and KDH) around the time of colonoscopy.

We also reviewed the clinical course of patients in medical records after acquiring biopsy specimens. We defined clinical recurrence as a change in prescription, bowel resection, fistulotomy, strictureplasty, stoma formation, CD-related hospitalization, or flare during the follow-up period [11]. CD-related hospitalization was defined as hospitalization because of complications including the following: CD-related surgery, hospitalization for nonsurgical CD-related events such as CD-related flares, hospitalization related to complications/extraintestinal manifestation of CD, and disease flare.

### Statistical analysis

Results are expressed as mean (standard deviation, SD) or median value (range). Continuous variables in 2 groups were compared using Student’s t-test or the Mann–Whitney test. IHC scores for Cyr61 expression in patients with CD were divided into tertiles: 1st, < 60 (n = 27); 2nd, 60–80 (n = 30) and 3rd, ≥ 80 (n = 26). We compared the clinical disease activities such as CDAI, SES-CD and CD-EIS according to tertiles of IHC score for Cyr 61 expression using Kruskall Wallis test. The association between Cyr61 expression and clinical recurrence was assessed using univariate and multivariable binary logistic regression. Odd ratio (OR) and *P* value are presented. Tests for trend were performed using the Cyr61 expression tertiles as ordinal variables in the corresponding logistic regression models. All statistical analyses were performed using SPSS 20.0 (SPSS Inc., Chicago, IL, USA). Two-sided *P* values < 0.05 were considered statistically significant.

### Ethical consideration

The study protocol was approved by the Ethics Committee of Chonnam National University Hospital (IRB No. CNUH-2020-121) and was conducted according to the Declaration of Helsinki and Good Clinical Practice guidelines.

## Results

### Comparison of Cyr61 expression in patients with CD and matched controls

**Fig. 1:**
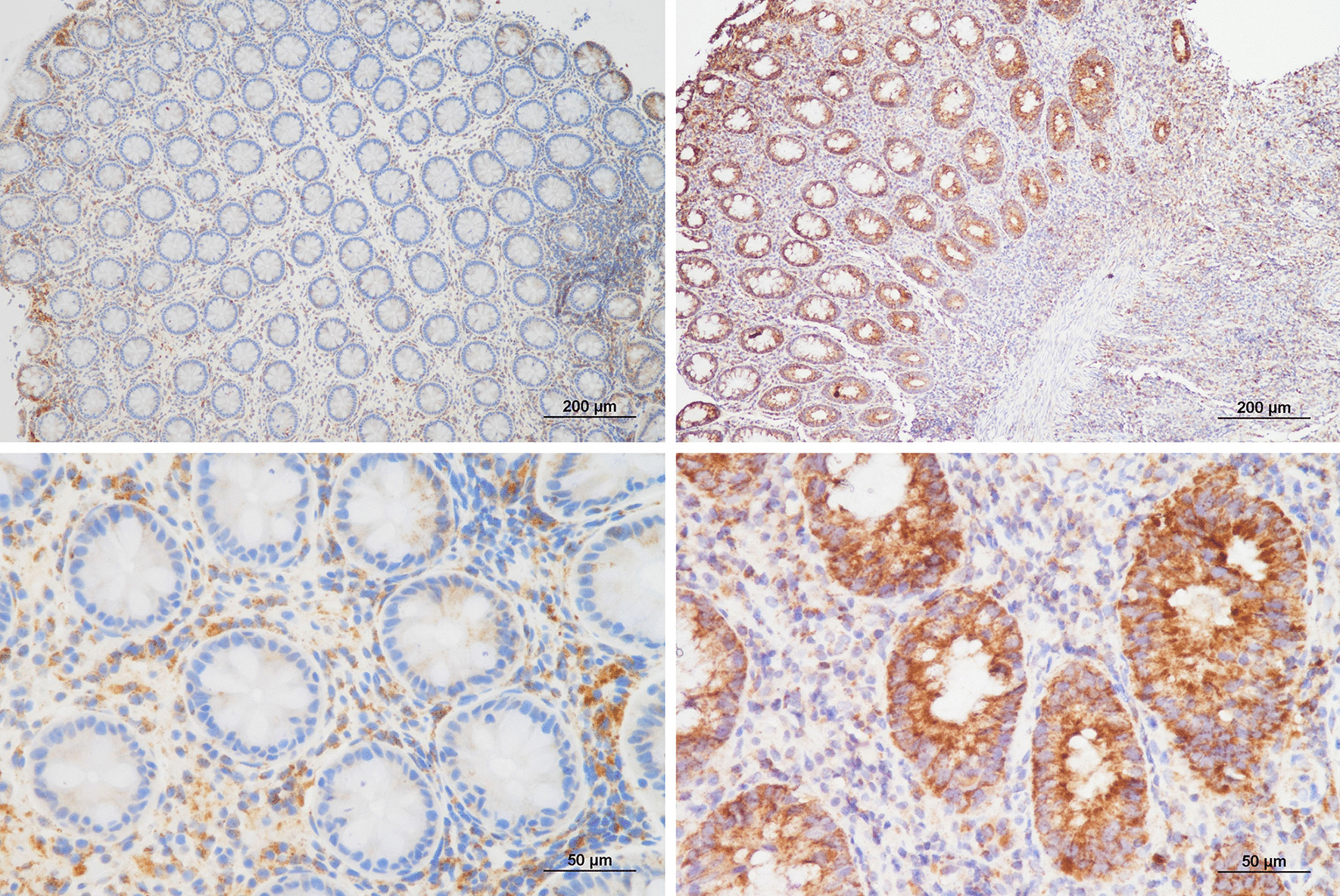
Representative histofigures of Cyr61 immunohistochemistry. **a** Control 1 of the Control group showed low Cyr61 expression. **b** Case 39 of the CD group displayed enhanced Cyr61 expression. **c** In the high-magnification view of the same site shown in panel **a**, little cytoplasmic staining with some membrane staining was observed. **d** Similarly, a high-magnification view of the same site in panel **c** showed strong cytoplasmic staining and highlighted cell borders

IHC of Cyr61 was performed on FFPE tissue blocks obtained from 43 patients with CD and 43 controls matched by propensity score for age and sex. Of 43 controls, two were excluded due to inadequate tissue blocks. Therefore, Cyr61 expression was analyzed in 43 patients with CD (36 with colonic tissue and 7 with ileal tissue) and 41 controls (all colonic tissue). The mean ages (SD) of patients with CD and controls were 34.8 (12.9) and 35.5 (12.2) years, respectively (*P* = 0.797). There were 31 men in both the patients with CD and control groups (*P* > 0.999). In patients with CD, mean inflammatory histologic score (SD) was 9.4 (1.7) in 36 colonic tissue and 5.7 (1.4) in 7 ileal tissue. Figure 1 showed representative images of control group (Fig. 1a, c) and patients with CD (Fig. 1b, d).

**Fig. 2:**
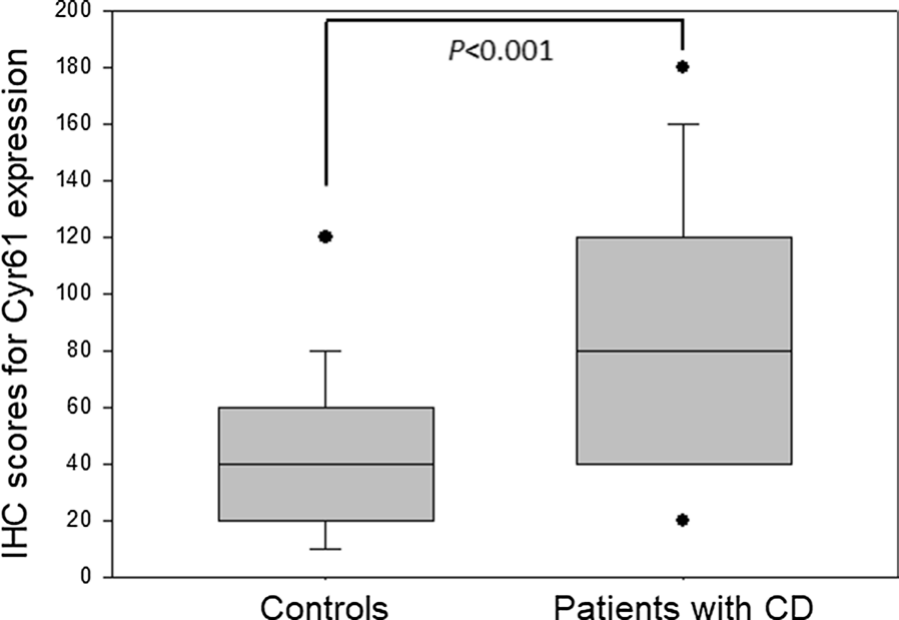
IHC scores of Cyr61 expression between control and Patients with CD. The Mean IHC scores for Cyr61 expression was higher in patients with CD (86.5) than in controls (46.1, *P* < 0.001). The upper and lower whiskers indicate the 90th and 10th percentiles

The IHC scores for Cyr61 expression was higher in patients with CD (86.5 ± 45.9) than in controls (46.1 ± 13.6, *P* < 0.001, Fig. 2). In detail, the Cyr61 staining area of patients with CD (48.3% ± 21.8%) was larger than that of controls (25.6% ± 13.6%, *P* < 0.001), while there was no significant difference in ‘high expression’ of Cyr 61 between patients with CD (34/43, 79.0%) and controls (32/41, 78.0%, *P* > 0.999).

### Expression of Cyr61 and pro-inflammatory genes and between inflamed and noninflamed mucosa in patients with CD

**Fig. 3:**
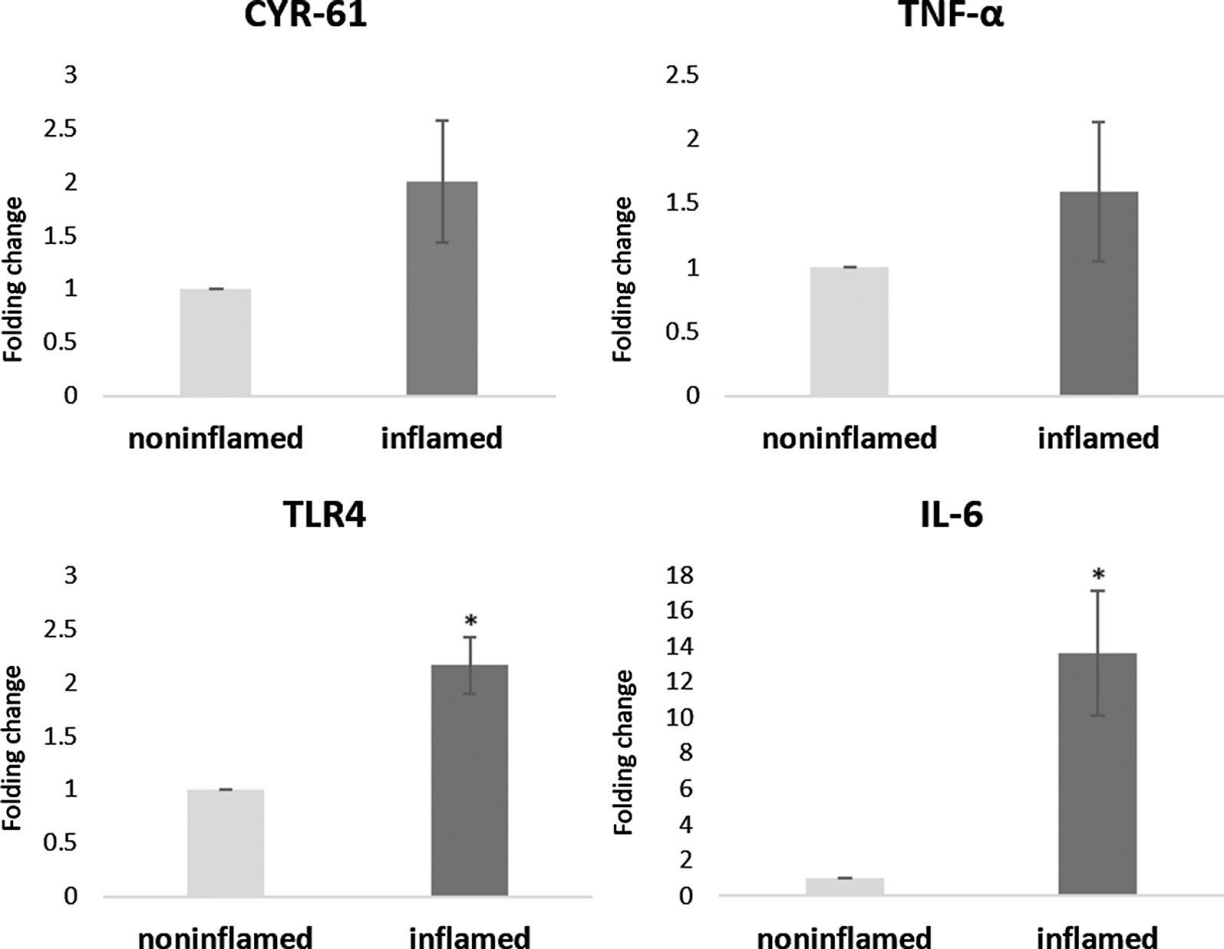
Expression of Cyr61 and pro-inflammatory genes between inflamed and noninflamed mucosa in patients with Crohn’s disease. The mRNA levels of IL-6^*^ (*P* < 0.01) and TLR-4^*^ (*P* < 0.01) in inflamed mucosa were significantly higher than those in non-inflamed mucosa. The mRNA levels of Cyr61 in inflamed mucosa was twofold higher than those in non-inflamed mucosa (*P* = 0.10)

To verify the degree of inflammation between the inflamed and noninflamed mucosa, we evaluated the mRNA levels of inflammatory genes. The mRNA levels of IL-6 (*P* = 0.006) and TLR-4 (*P* = 0.003) in inflamed mucosa were significantly higher than those in non-inflamed mucosa. Cyr61 mRNA levels were twofold higher, without significance, than those in non-inflamed mucosa (*P* = 0.096, Fig. 3).

### Association between IHC expression of Cyr61 and disease activity in patient with CD

Clinical characteristics of 83 patients with CD are shown in Table 1. There were 62 men (74.7%), and the mean age ± SD was 30.5 ± 10.7 years. The median values (range) of CDAI, SES-CD, and CDEIS were 106 (2–470), 4 (0.25–31.5), and 5.0 (1–36), respectively. There were no differences of CDAI (*P* = 0.620), SES-CD (*P* = 0.482) and CDEIS (*P* = 0.401) according to tertiles of IHC scores for Cyr61 expression.

**Table 1:** Baseline characteristics of 83 patients at the time of colonoscopy

	Patients without clinical recurrence (n = 54)	Patients with clinical recurrence (n = 29)	*P* value
Age, years., (mean ± SD)	31.6 ± 11.1	28.4 ± 9.9	0.21
Male, n (%)	40 (74.1%)	22 (75.9%)	0.86
Disease duration, years, median (range)	4.0 (0.0 ~ 15.0)	3.0 (0.0 ~ 12.0)	0.06^a^
Location of disease (Montreal classification), n (%)			0.54
Ileum (L1)	4 (7.4%)	2 (6.9%)	
Colon (L2)	2 (3.7%)	1 (3.4%)	
Ileocolon (L3)	48 (88.9%)	24 (82.8%)	
Concomitant UGI disease (L4)	0 (0.0%)	2 (6.9%)	
Medication at the time of colonoscopy, n (%)			
5-ASA	42 (77.8%)	19 (65.5%)	0.23
Systematic steroid	0 (0.0%)	2 (6.9%)	0.12^b^
Azathioprine/6-mercaptopurine	19 (35.2%)	5 (17.2%)	0.09
TNF-α antagonist	4 (7.4%)	3 (10.3%)	0.70^b^
CDAI, median (range)	100 (2–328)	171 (41–470)	< 0.01^a^
CDEIS, median (range)	4.0 (0.25–31.5)	4.0 (0.25–31.0)	0.50^a^
SES-CD, median (range)	5.0 (1.0–36.0)	5 (1–27)	0.33^a^

*SD* standard deviation, *5-ASA* 5-aminosalicylic acid, *CDAU* Crohn’s disease activity index, *CDEIS* Crohn’s disease index of severity, *SES-CD* simple endoscopic score for Crohn’s disease

^a^Man–Whitney U test

^b^Fisher’s exact test

### Association between IHC expression of Cyr61 and clinical course in patient with CD

Among 83 patients with CD, 29 (34.9%) had clinical recurrence during the follow-up period (median 19 months, range 11–26 months). There were 13 patients with a change in prescription, 2 with bowel resection, 6 with fistulotomy, 1 with strictureplasty, 1 with bowel resection and stoma formation, and 15 with CD-related hospitalization. There were no differences in age, sex, disease duration, locations of involvement, medication at the time of colonoscopy between patients with clinical recurrence and patients without clinical recurrences (Table 1, all *P* > 0.05). Median CDAI score in patients with clinical recurrence (171) was higher than that in patients without clinical recurrence (100, *P* < 0.01).

**Fig. 4:**
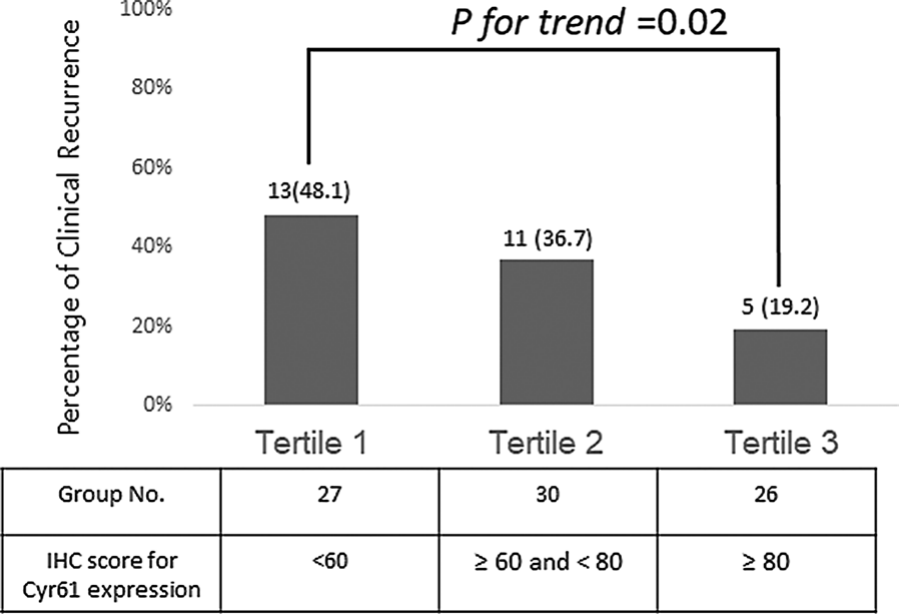
Clinical recurrence rates according to tertiles of Cyr 61 expression in patients with CD. Data are presented as frequencies (percentages)

The IHC scores for Cyr61 expression was lower in patients with clinical recurrence (68.3 ± 34.9) than in patients without clinical recurrence (92.2 ± 49.3, *P* = 0.01). When the patients with CD were stratified into tertile groups according to IHC scores for Cyr61 expression, clinical recurrence rates tended to be lower in patients with high IHC scores for Cyr61 expression (*P* for trend = 0.02, Fig. 4). The ORs for clinical recurrences according to tertiles of Cyr61 expression are shown in Table 2. Compared with tertile 1 of Cyr61 expression, tertile 3 of Cyr 61 expression was associated with reduced risk of clinical recurrence (OR 0.43, 95% CI 0.20–0.92) after adjustment for age, sex and CDAI at the time of colonoscopy (Table 2).

**Table 2:** Odds ratio for clinical recurrence according to Cyr61 expression tertiles

Variables	IHC scores for Cyr61 expression	No. of patients	Prevalence of clinical recurrence	Unadjusted		Adjusted*	
				OR (95% CI)	*P* value	OR (95% CI)	*P* value
Tertile 1	< 60	27	48.1%	1.0		1.0	
Tertile 2	≥ 60 and < 80	30	36.7%	0.62 (0.25–1.80)	0.38	0.59 (0.18– 1.88)	0.37
Tertile 3	≥ 80	26	19.2%	0.26 (0.08–0.89)	0.03	0.43 (0.20– 0.92)	0.03
P for trend				0.03			0.02

^*^Adjusted for age, sex and CDAI; OR, odd ratios

## Discussion

In this study, we showed a significant increase in the colonic mucosal expression of Cyr61 in patients with CD compared to that in controls. Colonic mucosal expression of Cyr61 was inversely associated with clinical recurrence in patients with CD.

Several studies showed that the expression of Cyr61 was increased in chronic inflammation models [12–14]; this was also increased in LPS-treated macrophages [12] and in a DSS-induced colitis model, especially during the recovery phase [15]. Su et al. showed that Cyr61 expression was elevated by IL-8 stimulation in gastric cancer cell lines [14]. In skin wound healing and chronic inflammatory liver injuries, Cyr61 played a role in reducing fibrosis during the maturation phase of tissue repair by triggering cellular senescence in activated myofibroblasts [16–18]. In an experimental model of alcoholic hepatitis, Cyr61 exacerbated apoptosis of hepatocytes [18]. Cyr61 was also known to have angiogenic activity in a model of bone fracture repair [19]. Recent studies reported the involvement of Cyr61 in the pathogenesis of chronic inflammatory diseases such as rheumatoid arthritis [8, 20], psoriasis vulgaris [21], Sjogren’s syndrome [22], and SLE [7].

In this study, we demonstrated the increased mucosal expression of Cyr61 in patients with CD. To minimize the effects of age and sex on Cyr61 expression, we used propensity score-matching analysis for selection of patients with CD and controls. The IHC expression of Cyr61 protein in colonic mucosa from patients with CD was significantly higher than that in colonic mucosa from controls. Previous study suggested that Cyr61 protein expression was observed in only surface epithelial cells of the normal colon, whereas Cyr61 protein expression was observed in the entire mucosal epithelium of DSS-induced colitis mouse models [13]. Likewise, whereas the intensity of Cyr61 expression of patients with CD and controls was not different, the area of Cyr61 expression was higher in patients with CD.

Cyr61 has been known to contribute to inflammatory damage by inducing pro-inflammatory cytokine expression in macrophages and enhancing the cytotoxicity of TNF family cytokines [13, 16, 22]. Cyr61 was associated with upregulated expression of TNF-α, IL-6, and IL-17 in patients with SLE [7]. Previous study showed that IL-6 was stimulated by Cyr61, and downregulation of Cyr61 led to reduced IL-6 in fibroblast-like synoviocytes in RA [23]. In this study, we explored the colonic mucosal expression of Cyr61 in inflamed and non-inflamed mucosa from patients with CD. Cyr61 tended to be increased in inflamed mucosa with higher proinflammatory gene expression including IL-6 and TLR-4.

Cyr61 is also capable of wound healing and tissue repair. Recently, Cyr61 has been reported to opsonize gram negative and gram positive bacteria to accelerate bacterial clearance [24]. Several studies demonstrated that administration of Cyr61 protein accelerated epithelial repair in murine colitis models, human lung epithelial cells and cutaneous wound [13, 25, 26]. Du et al. showed that upregulation of Cyr61 during the early stage of wound healing and treatment of recombinant human Cyr61 promoted reepithelialization [26]. In line with these finding, it is plausible that variable stress factors may upregulate Cyr61 expression, which may activate inflammatory responses contributing tissue repair in pathomechanism of CD. Therefore, lower Cyr61 expression may be associated with delayed tissue repair leading to worse prognosis. In this present study, we didn’t perform the functional experiments about the role of Cyr 61 for tissue repair after inflammation in patients with CD. This is the limitation of the current study. Another limitation is that we retrieved the CDAI score, which was calculated and written on the chart by colonoscopist from the collection of daily status for past 7 days. CDAI is the most frequently used index for evaluation of disease activity and the calculation of the CDAI is based on a diary filled in by the patients for 7 days before evaluation [27]. Therefore, we need to perform a well-designed prospective study whether Cyr 61 may play a role in activating inflammatory responses and contributing to wound healing and tissue repair in patients with CD.

## Conclusions

Cyr61 mucosal expression in endoscopic biopsy specimens from patients with CD was inversely associated with clinical course. Future study need to be considered to evaluate whether Cyr 61 may play a role in activating inflammatory responses and contributing to wound healing and tissue repair in patients with CD.

## Data Availability

The datasets generated during and/or analyzed during the current study are available from corresponding author under reasonable request.

## Abbreviations

Cyr61: Cysteine-rich angiogenic inducer 61
CD: Crohn’s disease
IHC: Immunohistochemistry
SLE: Systemic lupus erythematosus
CDAI: Crohn’s disease activity index
SES-CD: Simple endoscopic score of Crohn’s disease
CDEIS: Crohn’s disease endoscopic index of severity
